# Role of the ER/NO/cGMP Signaling Pathway in the Promotion of Osteogenic Differentiation of Rat Bone Marrow Mesenchymal Stem Cells by* Actaea racemosa* Extract

**DOI:** 10.1155/2016/2615620

**Published:** 2016-11-15

**Authors:** Shenlan Yang, Yanping Zhou, Bo Shuai, Rui Zhu, Wei Xu, Yanran Wu, Danfang Deng, Yingying Luo

**Affiliations:** ^1^Department of Integrated Traditional Chinese and Western Medicine, Union Hospital, Tongji Medical College, Huazhong University of Science and Technology, Wuhan, Hubei 430022, China; ^2^Department of Traditional Chinese Medicine, Huazhong University of Science and Technical Hospital, Wuhan, Hubei 430074, China; ^3^Hubei University of Traditional Chinese Medicine, Wuhan, Hubei 430065, China

## Abstract

*Purpose*/*Objective*. To investigate the effect of* Actaea racemosa* (AR) extract on* in vitro* osteogenic differentiation of rat bone marrow mesenchymal stem cells (BMSCs) via the ER/NO/cGMP signaling pathway.* Methods*/*Materials*. Rat BMSCs were treated with osteogenic differentiation-inducing medium containing AR; estrogen receptor antagonist, ICI 182,780 (10^−6^ mol/L); and nitric oxide synthase inhibitor, L-nitro arginine methyl ester (L-NAME, 6 × 10^−3^ mol/L). Markers of osteogenic differentiation (alkaline phosphatase [ALP] activity, osteocalcin secretion, and calcium ion deposit levels) and the levels of key signaling molecules (nitric oxide synthase [NOS], nitric oxide [NO], and cyclic guanosine monophosphate [cGMP]) were assessed.* Results*. AR (10^−1^–10^−6^ g/L) increased ALP activity in a dose-dependent manner, and the highest ALP, osteocalcin, and osteoprotegerin activities were achieved at an AR concentration of 10^−4^ g/L. Therefore, the concentration of 10^−4^ g/L was used for promoting osteogenic differentiation of BMSCs in subsequent analyses. At this concentration, AR increased the levels of NO and cGMP, and such effects could be blocked by the estrogen receptor antagonist (ICI 182,780) and nitric oxide synthase inhibitor (L-NAME).* Conclusion*. AR induced osteogenic differentiation of rat BMSCs through the ER/NO/cGMP signaling pathway. This finding provides the theoretical foundation for the mechanism of AR in the treatment of postmenopausal osteoporosis.

## 1. Introduction

Postmenopausal osteoporosis (PMOP) is a prevalent disease that affects 50% of women around the age of 60 mainly due to estrogen deficiency. Estrogen replacement therapy is effective in preventing and treating PMOP, but its risks outweigh its benefits [1]. Therefore, several researchers are searching for alternative medications for treating PMOP.

*Actaea racemosa* (AR) extract has long been used to treat perimenopausal syndrome because it does not contain estrogen but has effects to alleviate menopausal symptoms, particularly hot flushes, night sweats, and correlated sleep disturbances [2, 3]. In addition, its safety and efficacy have already been confirmed [2–11]. Clinical tests have shown that AR influenced the levels of bone turnover markers in the serum of patients with PMOP, demonstrating its protective effect on the bone [12, 13]. Experimental studies in animals demonstrated that AR can promote healing of fracture and can reduce the loss in bone mass in osteoporotic rats caused by ovariotomy, whereas it has no effect in the uterus [14–16]. However, the potential mechanism of AR for treatment of PMOP is still unknown.* In vitro* experiments showed that AR increased the levels of osteoprotegerin (OPG), alkaline phosphatase (ALP), and osteocalcin (OC) secreted by osteoblasts and promoted the formation of mineralized bone nodules as well as the expression of* Runx2* and* OC* in MC3T3-E1 precursor cells [17, 18]. Moreover, these effects could be blocked by the estrogen receptor (ER) antagonist (ICI 182,780, 10^−6^ mol/L). Interestingly, bone tissue is rich in estrogen-*β*-receptors (ER-*β*) in contrast to ER-*α*-rich tissues such as breast or endometrium. Therefore, this was indicating that AR has a positive effect on the bone partially through ER signaling [17, 18].

Recent studies have shown that the nitric oxide (NO)/cyclic guanosine monophosphate (cGMP) signaling pathway is closely related to bone metabolism. NO inhibits bone resorption by osteoclasts and promotes differentiation of osteoblasts [19]. In addition, NO produced by osteoblasts activates guanylate cyclase to increase the formation of cGMP and further accelerates its stimulating effect on osteoblasts [20]. Other active ingredients from Chinese medicinal herbs, specifically, genistein, resveratrol, and icariin, also promote osteogenic differentiation of bone marrow mesenchymal stem cells (BMSCs) through this signaling pathway [19–21]. To the best of our knowledge, no study has investigated whether AR participates in bone metabolism through this signaling pathway. In this study, we investigated the effect of AR on markers of osteogenic differentiation, particularly ALP activity, calcium ion deposit levels, and key signaling pathway molecules, nitric oxide synthase (NOS), NO, and cGMP, in order to detect whether the signaling through the ER/NO/cGMP pathway is a predominant mechanism utilized by AR for treatment of PMOP.

## 2. Materials and Methods

### 2.1. Experimental Animals

Specific pathogen free- (SPF-) grade adult male Sprague Dawley (SD) rats (*n* = 8) weighing around 120 g were obtained from the Experimental Animal Center, Tongji Medical College, Huazhong University of Science and Technology (HUST), under the Animal Quality Certificate Number SCXK 2010-0009. Notably, all experiments were performed in accordance with HUST's ethical guidelines for animal care as well as the guidelines set by the World Health Organization, and the experimental protocols were all approved by the animal care committee of HUST.

### 2.2. Reagents

AR reference standard (USP, USA, Cat. Number 1076206, 100 mg) was dissolving with 70% ethanol/water (1 mL) and then using an osteogenic induction medium (10% activated carbon-absorbed bovine serum [BI, Israel], DMEM/F12 medium without phenol red [FBS, Australian origin], 1% penicillin-streptomycin solution [Gibco, USA], 10^−8^ mol/L dexamethasone, 10^−2^ mol/L sodium *β*-glycerophosphate, and 50 *μ*g/mL vitamin C [Sigma, USA]) to supplement with varying concentrations of 10^−1^–10^−6^ g/L. The following were obtained: DMEM/F12 medium with phenol red, fetal bovine serum (FBS, Australian origin); phosphate buffer solution (PBS), 0.25% pancreatin, and BCA Protein Assay Kit (Sigma, USA); ER antagonist (ICI 182,780, Selleck, USA); ALP Assay Kit, OC Assay Kit, OPG Assay Kit, NO Assay Kit, NOS Assay Kit, Calcium Assay Kit, rat cGMP ELISA Kit, and nitric oxide synthase inhibitor L-NAME (Nanjing Jiancheng Bioengineering Institute); anti-rat CD29-PE, anti-rat CD90-FITC, anti-rat CD45-APC, and anti-rat CD11b-PE-Cy7 (eBioscience, USA); Trizol reagent (Invitrogen, USA); trichloromethane, anhydrous ethanol, and isopropanol (Sinopharm Chemical Reagent Co., Ltd., China); HyPure™ Molecular Biology Grade Water (HyClone, USA); Revert Aid First Strand cDNA Synthesis Kit (Thermo, USA); Fast Start Universal SYBR Green Master (Rox) (Roche, Switzerland). All primers were synthesized by Invitrogen Biotechnology Co., Ltd. (Table 1).

### 2.3. Experimental Methods

#### 2.3.1. Extraction and Maintenance of Rat BMSCs

A previously described method was applied for cell extraction and maintenance [22]. SD rats were euthanized and the thigh and shank bones were quickly isolated. The osteoepiphyses at the ends of the bones were removed and the bone marrow was completely flushed. Cell colonies were dispersed by blowing. The cells were then filtered through a 200-mesh screen, and the resulting filtrate was a single-cell suspension. The cell density was adjusted to 1 × 10^9^/L, and a 3 mL cell suspension was inoculated into each cell culture flask containing 10% FBS. This was incubated at 37°C, 5% CO_2_, and saturated humidity for 24 h, after which the culture medium was replaced. Cells were digested with 0.25% pancreatin and subcultured at approximately 80% confluence. Cell surface antigen expression and cell purity were determined by flow cytometry using the third subculture of cells.

#### 2.3.2. Identification of the Rat BMSC Phenotype

The third subculture of rat BMSCs (1 × 10^9^/L) was centrifuged for 5 min at 1500 rpm, and the supernatant was discarded. Cells were blocked in BSA solution at 4°C for 1 h and centrifuged as before. The cells were stained with anti-rat CD90-FITC, anti-rat CD29-PE, anti-rat CD45-APC, and anti-rat CD11b-PE-Cy7 in the dark for 20 min. The cells were then washed with PBS to remove unbound antibodies and immediately analyzed on a flow cytometer to determine antigen expression and cell purity.

#### 2.3.3. Determination of the Optimal AR Concentration for Promoting Osteogenic Differentiation of BMSCs

When cells attained approximately 80% confluence, the culture medium was changed to an osteogenic induction medium, supplemented with varying concentrations of AR (10^−1^ g/L, 10^−2^ g/L, 10^−3^ g/L, 10^−4^ g/L, 10^−5^ g/L, 10^−6^ g/L, 0 [0.1% ethanol, v/v]), and the cells were incubated for 8 days. The osteogenic induction medium supplemented with varying concentrations of AR was changed every two days. Cells were harvested on day 8 and the ALP, OC, and OPG activities were determined (the specific procedures were described previously by Viereck et al. [17]). By comparing the ALP, OC, and OPG activities of different cultures, the optimal AR concentration was determined to be 10^−4^ g/L, which was used in subsequent analyses (see Results and Figure 2).

#### 2.3.4. Detection of Osteogenic Differentiation Markers and Related Signaling Pathway Molecules

When cells were approximately 80% confluent, the normal culture medium was replaced with the osteogenic induction medium, and cells in the AR (10^−4^ g/L); AR + ICI 182,780 (10^−6^ mol/L); AR + L-NAME; ICI 182,780 (6 × 10^−3^ mol/L) [18]; L-NAME; and control (0.1% ethanol [v/v]) groups were treated for culture medium change as mentioned earlier. Total NOS and ALP activities, NO, cGMP, OC, and OPG contents [17], calcium ion deposit and ALP, eNOS, and iNOS mRNA expression levels were determined.

#### 2.3.5. Detection of ALP, OC, and OPG Activities

Cells were collected on day 8 after treatment, lysed as previously described, and centrifuged [22]. The supernatant (30 *μ*L) was evenly mixed with detection reagent, and color development was measured as absorbance at 520 nm using a microplate reader. The total secreted protein content was determined using a BCA Protein Assay Kit. The secreted ALP activity (expressed in U/g prot), OC activity (expressed in ng/mL), and OPG activity (expressed in ng/L) in each group [17] were calculated according to the absorbance, sample amount, and protein concentration of the sample to be tested in each group.

#### 2.3.6. Detection of Calcium Ion Deposit Levels

Cells were collected on day 12 after treatment, lysed as previously described, and centrifuged. The supernatant (50 *μ*L) was evenly mixed with detection reagent and color development was measured as absorbance at 610 nm using a microplate reader. The total secreted protein content was determined using a BCA Protein Assay Kit. The Ca^2+^ content in each group, expressed in mmol/g prot, was calculated according to the absorbance, concentration of standard solution, and protein concentration of the sample to be tested in each group.

#### 2.3.7. Detection of Total NOS Activity

Cells were harvested on day 8, lysed with 0.2% Triton X-100 in an ultrasonic cell crusher, and centrifuged. The supernatant was then collected. Following the manufacturer's instructions for the nitric oxide synthase assay, 100 *μ*L of supernatant was evenly mixed with the detection reagent, and color development was measured as absorbance at 530 nm using a microplate reader. The total protein content was determined using a BCA Protein Assay Kit. The total NOS activity in each group, expressed in U/mg prot, was calculated based on the absorbance, sample amount, and protein concentration in each group.

#### 2.3.8. Determination of NO Content in the Supernatant of Cells

The NO content in the supernatant of cells was determined using the nitric oxide assay kit, according to the manufacturer's instructions. The NO content in the supernatant in each group was calculated according to synthase activity (*μ*mol/L) and was calculated based on corresponding absorbance and reference standard concentrations.

#### 2.3.9. Determination of cGMP Content

The cGMP content was determined using the rat cGMP ELISA Assay Kit. Cells were lysed as described and centrifuged, and the supernatant was collected. Following the manufacturer's instructions, 40 *μ*L of supernatant was added to a 96-well plate and allowed to react, and the absorbance at 450 nm was measured using a microplate reader. A standard curve was obtained by plotting the concentrations of the standard solution against corresponding absorbance. The sample concentration, expressed in nmol/L, was calculated according to the absorbance of the sample to be tested.

#### 2.3.10. Fluorescence-Based Real-Time Polymerase Chain Reaction (qPCR)

Treated cells were collected and total RNA was extracted using TRIzol reagent. The Revert Aid First Strand cDNA Synthesis Kit was used to reverse-transcribe the RNA into corresponding cDNA. Using cDNA as the template, 2x qPCR Mix and 7.5 *μ*M gene primers were added for PCR amplification under specified cycling conditions on a 7300 real-time PCR system. The Fast Start Universal SYBR Green Master (Rox) was used and the expression levels of* ALP*,* eNOS*, and* iNOS* were determined. The C_T_ values (ΔΔC_T_ method) were calculated and analyzed as previously described [23]. The primer sequences are given in Table 1.

#### 2.3.11. Statistical Analysis

Statistical analyses were performed by analysis of variance (ANOVA) using SPSS software (version 20.0, SPSS, Inc., Chicago, IL, USA). All test data were expressed as mean ± standard deviation, and *P* < 0.05 was considered statistically significant.

## 3. Results

### 3.1. Morphological Observation and Phenotypic Characterization of Rat BMSCs

**Figure 1:**
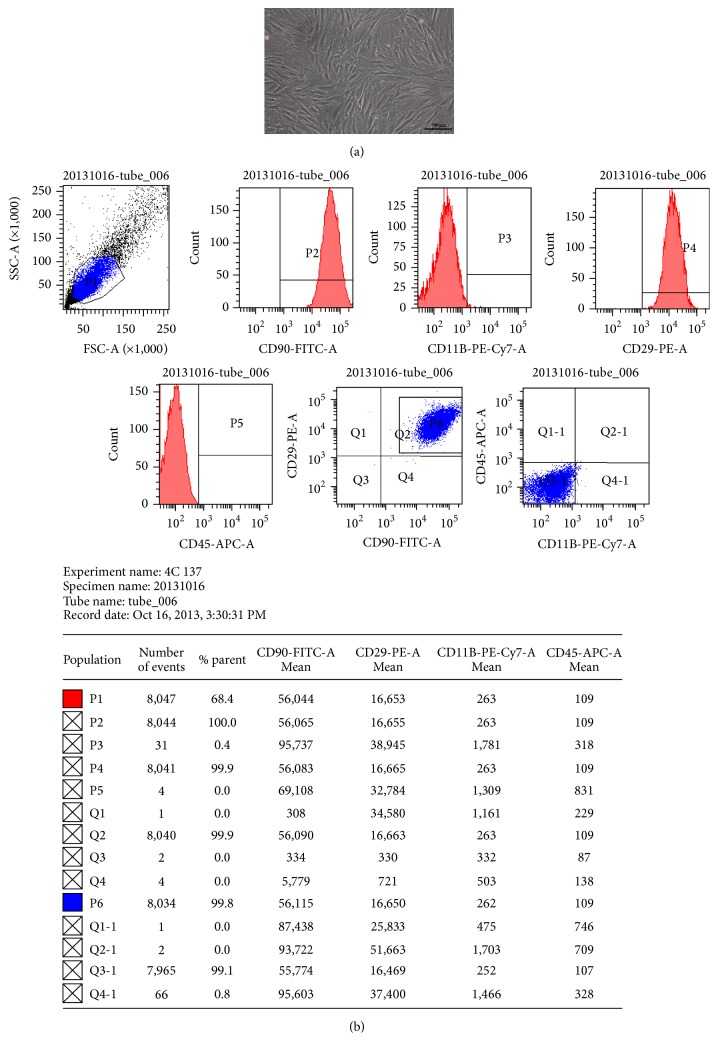
(a) Morphological observation (200x) of third-subculture rat BMSCs and (b) expression levels and purity of surface markers of third-subculture rat BMSCs. (a) The third-subculture cells had a long spindle-shaped morphology and were densely arranged in a swirling pattern, demonstrating the typical morphology of rat BMSCs. (b) Fluorescent-labeled CD90, CD29, CD11b, and CD45 were incubated with BMSCs, and the labeled cells were detected on a flow cytometer.

Primary cells were cultured for 24 h followed by changing the culture medium. After 9–12 days of incubation, cells grew to approximately 80% confluence in a monolayer with cells arranged in specific directions. During subculturing, cells grew considerably faster and reached 80–90% confluence within 5-6 days. Cells at the third subculture had a long spindle-shaped morphology and were densely arranged in a swirling pattern (Figure 1(a)). Flow cytometry analysis demonstrated that these cells expressed CD90 (100%) and CD29 (99.9%) but did not express CD45 (0%) and CD11b (0.4%) (Figure 1(b)).

### 3.2. AR Induced Osteogenic Differentiation of BMSCs in a Dose-Dependent Manner

**Figure 2:**
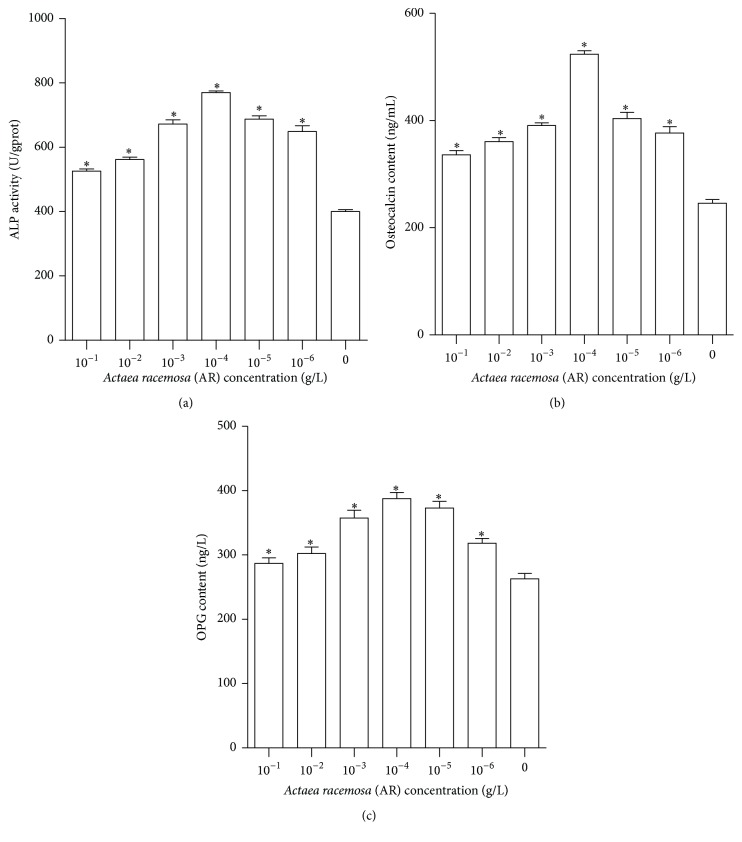
AR increased osteogenic differentiation of rat BMSCs in a dose-dependent manner. The ALP activity and OC and OPG contents were detected after 8 days of treatment with varying AR concentrations. (a) ALP activity for varying AR concentrations. (b) OC content for varying AR concentrations. (c) OPG content for varying AR concentrations. ^*∗*^ *P* < 0.001 versus control.

BMSCs were treated with 10^−1^–10^−6^ g/L AR. As shown in Figure 2, AR increased ALP, OC, and OPG activities in a dose-dependent manner to a level significantly higher than the control (0.1% ethanol [v/v]) group (*P* < 0.001). AR did not stimulate highest activities of ALP, OC, and OPG at concentration of 10^−1^ g/L (highest doses of AR) and 10^−6^ g/L (lowest doses of AR) but at 10^−4^ g/L, which showed a significantly increased stimulating potentiate bone forming activity. Therefore, the concentration of 10^−4^ g/L was used for promoting osteogenic differentiation of BMSCs in subsequent analyses.

### 3.3. L-NAME and ICI 182,780 Blocked the Positive Effect of AR on Osteogenic Differentiation Markers and ER/NO/cGMP Signaling in Rat BMSCs

#### 3.3.1. Detection of ALP Activity, mRNA Expression, and Calcium Ion Deposit Level

**Figure 3:**
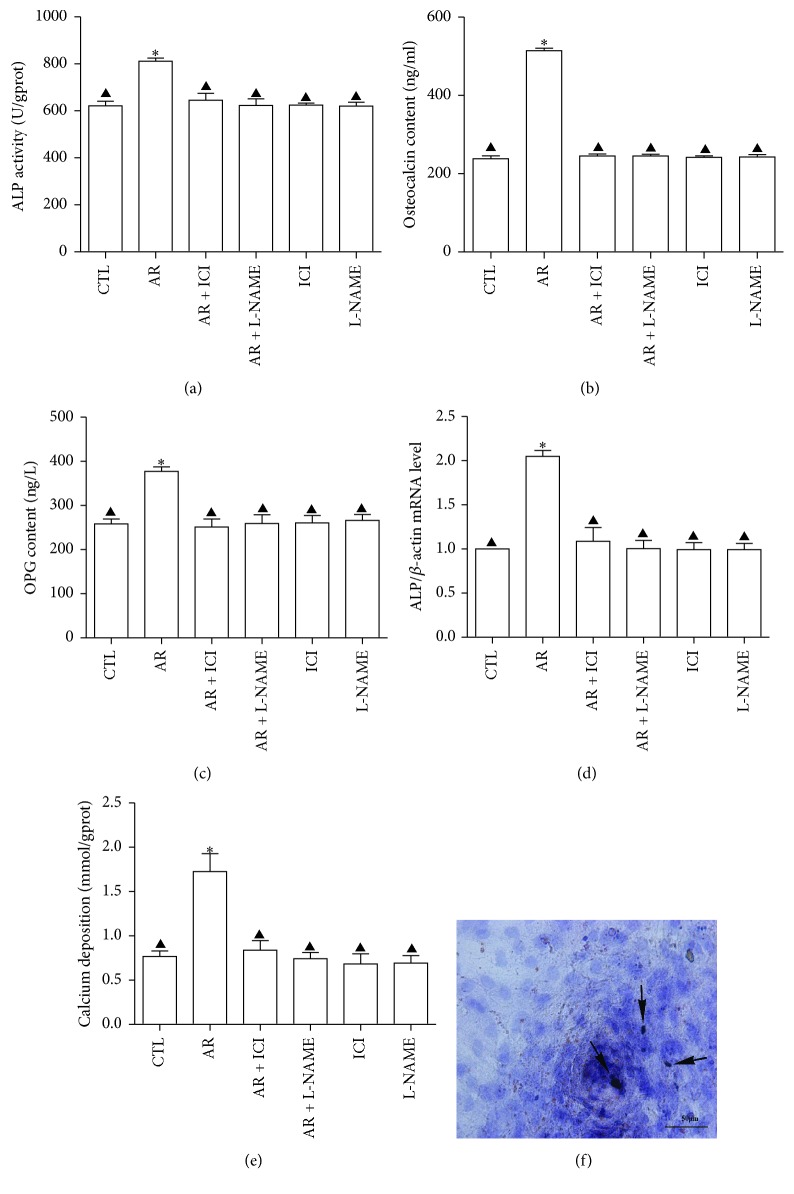
Induction of osteogenic differentiation of rat BMSCs by adding AR; ICI 182,780; and L-NAME to the osteogenic induction medium for 8 days. ALP activity, OC and OPG contents, and ALP mRNA expression and calcium ion deposit levels were determined. (a) ALP activity for indicated groups. (b) ALP mRNA expression levels for various groups. (c) OC content for indicated groups. (d) OPG content for various groups. (e and f) Levels of calcium ion deposits for various groups. ^*∗*^ *P* < 0.001 versus control; ^▲^ *P* < 0.001 versus AR. Concentration of AR was 10^−4^ g/L; ICI 182,780 was 10^−6^ mol/L; L-NAME was 6 × 10^−3^ mol/L; and the control was 0.1% ethanol (v/v).

After 8 days of AR treatment, a significant increase in ALP activity and OC and OPG contents was observed as compared to those in the control group (*P* < 0.001, Figures 3(a)–3(c)). The values of *β*-actin expression were 16.065 ± 0.213, 16.167 ± 0.204, 16.158 ± 0.209, 16.081 ± 0.201, 16.146 ± 0.215, and 16.131 ± 0.212 in the control group, AR group, AR + ICI 182,780 group, AR + L-NAME group, ICI 182,780 group, and L-NAME group, respectively. They were not significantly different from each other (*F* = 1.38,  *P* > 0.05); therefore, it could serve as housekeeping gene. AR increased the ALP mRNA expression 2.05 times as compared to that in the control group (Figure 3(d)). The positive effect of AR on ALP, OC, and OPG activities and ALP mRNA expression was blocked by ICI 182,780 and L-NAME, whereas cells treated with either of the blocking agents alone exhibited no difference in ALP, OC, and OPG levels and ALP mRNA expression as compared to those in the control group (Figures 3(a)–3(d)).

AR also significantly increased the levels of calcium ion deposit after 8 days of treatment as compared to that in the control group (*P* < 0.001). This effect was blocked by ICI 182,780 and L-NAME treatment. Similarly, cells treated with either ICI 182,780 or L-NAME alone exhibited no difference in calcium ion deposit as compared to that in the control group (Figures 3(e) and 3(f)).

#### 3.3.2. Determination of TNOS Activity, eNOS and iNOS mRNA Expression, and NO and cGMP Contents

**Figure 4:**
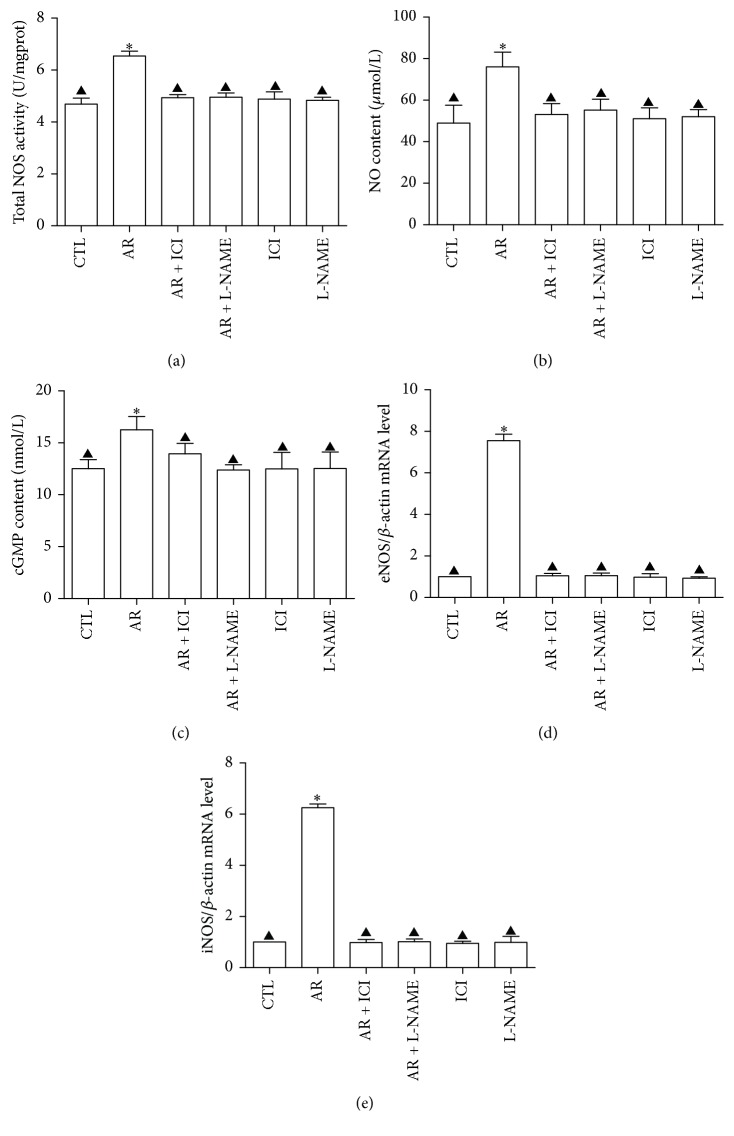
Induction of osteogenic differentiation of rat BMSCs by adding AR; ICI 182,780; and L-NAME to the osteogenic induction medium for 8 days. The TNOS activity, cGMP content, eNOS and iNOS mRNA expression levels, and the NO content in the supernatant of cells were determined. (a) TNOS activity. (b) NO content in the supernatant of cells. (c) cGMP levels. (d) eNOS mRNA expression level. (e) iNOS mRNA expression level. ^*∗*^ *P* < 0.001 versus control; ^▲^ *P* < 0.001 versus AR. Concentration of AR was 10^−4^ g/L; ICI 182,780 was 10^−6^ mol/L; L-NAME was 6 × 10^−3^ mol/L; and the control was 0.1% ethanol (v/v).

AR significantly increased TNOS activity and NO and cGMP contents as compared to those in the control group, after 8 days of stimulation (*P* < 0.001) (Figures 4(a)–4(c)). AR increased eNOS and iNOS mRNA expression 7.55 times (Figure 4(d)) and 6.25 times (Figure 4(e)), respectively, as compared to those in the control group. The effects of AR could be blocked by ICI 182,780 and L-NAME, but not by either of the blocking agents alone (Figure 4).

## 4. Discussion

The safety and efficacy of AR in the treatment of perimenopausal syndrome have been confirmed [2, 3], but its potential treatment mechanism is still unknown. It is widely recognized that AR activating the neurotransmitter-based effects [24–27], rather than the estrogen activity [28, 29], is an important mechanism for alleviating menopausal symptoms, such as hot flashes and night sweats. Numerous studies have also confirmed that AR does not have a stimulating effect on the levels of blood estrogen and progestin or estrogen target organs such as the uterus, ovaries, breast, and blood vessels [9, 22, 30]. However, studies on the effect of AR on the skeletal system revealed that AR promotes the secretion of OPG and OC by human osteoblasts, increases the OPG/RANKL ratio, and promotes the formation of mineralized nodules in MC3T3-E1 preosteoblasts, all of which could be blocked by ICI 182,780 [17, 18]. In a preliminary study, a concentration of 10^−1^ to 10^−6^ g/L AR was shown to increase ALP, OC, and OPG activities in a dose-dependent manner. In this study, ALP activity, OC content, and OPG content, the three markers of osteogenic differentiation, reached the highest level at an AR concentration of 10^−4^ g/L, which was selected as the optimal concentration for osteogenic differentiation of BMSCs. This was similar to another cell culture study; the intermediate (500 ng/mL) concentration of AR was also found to be the largest enhanced bone nodules formation [18].

eNOS can be expressed at low levels in osteoblasts, osteoclasts, and marrow stroma cells. Studies show that eNOS is an indispensable molecule in the process, whereby 17*β*-estradiol promotes the functions of osteoblasts through the ER receptor signaling pathway [31]. iNOS is expressed at high levels in osteoblasts and bone marrow cells [32]. eNOS promotes the proliferation and differentiation of cells by generating NO at low concentration, whereas iNOS induces apoptosis by generating NO at high concentration [33]. In this study, we observed that TNOS activity was significantly higher in the AR group than that in the control; AR + ICI 182,780; and AR + L-NAME groups. eNOS and iNOS mRNA expression levels were significantly higher in the AR group than those in the control; AR + ICI 182,780; and AR + L-NAME groups. As compared to the control group, the AR group demonstrated significantly increased NO release. This was in agreement with the positive effect of AR on ALP activity and calcium ion deposit level, suggesting that the effects of AR on TNOS, eNOS, and iNOS mRNA as well as NO expression could be blocked by the nonselective NOS inhibitor L-NAME or ER antagonist ICI 182,780. This indicates that the regulating effect of AR on NO is at least partially mediated by ER, and NO promotes the differentiation of BMSCs into osteogenic cells. The results of this study also revealed that, under the same conditions for induction of osteogenic differentiation, BMSC generated two enzymes, eNOS and iNOS, and eNOS mRNA expression levels were higher than the iNOS mRNA expression levels in the AR group. This suggests that certain proinflammatory cytokines that induce iNOS production might be generated during the osteogenic induction process, and further studies are needed to confirm this speculation. Recent studies revealed that iNOS generates high concentration of NO to induce both apoptosis and autophagy in osteoblast, which appears to be a protective mechanism against apoptosis through AMPK activation [34]. AR may enhance this autophagy function to counteract iNOS-induced apoptosis. Understanding the specific mechanism requires further investigation.

cGMP plays an important role in the proliferation and differentiation of osteoblasts. Increased NO levels activate soluble guanylate cyclase to increase the cGMP content and accelerate the differentiation and mineralization of osteoblasts through the action of cGMP [35–37]. Here, we observed that, as compared to the control group, the AR group exhibited significantly increased cGMP content, which was in agreement with the increase in the NO release and the positive effect of AR on ALP activity and calcium ion deposition in BMSC cells. The NOS inhibitor L-NAME or ER antagonist ICI 182,780 could block this promotional effect of AR. This suggests that AR plays a very important role in promoting the differentiation of BMSCs into osteoblasts through the ER/NO/cGMP signaling pathway, which provides new information for future studies on the protective action of AR-based herbal medicinal products on bone tissues.

## 5. Conclusions

This study contributes important expansion of recent experiments on possible clinical application of AR for preventing PMOP. These experiments highlight the use of AR to induce the passage and differentiation of BMSCs isolated from osteoporotic rat into osteoblast-like cells. AR at 10^−9^ mol/L increases the concentration of NO and cGMP to the highest levels, and these effects could be blocked by the estrogen receptor antagonist (ICI 182,780) and NOS inhibitor (L-NAME). This suggests that AR plays a very important role in promoting the differentiation of BMSCs into osteoblasts through the ER/NO/cGMP signaling pathway. While these findings provide a secure foundation, additional work is warranted in order to fully understand the role of this herbal medicine in osteoporotic differentiation of BMSCs.

## Figures and Tables

**Table 1:** Primer sequences for real-time fluorescence-based quantitative PCR.

Gene	Genomic library code	Primer sequence	Sequence length/bp
eNOS	NM_021838.2	Forward 5′-ACTATGGCAACCAGCGTCCT-3′	143
		Reverse 5′-CGCAATGTGAGTCCGAAAATG-3′	
iNOS	NM_012611.3	Forward 5′-CTCACTGTGGCTGTGGTCACCTA-3′	101
		Reverse 5′-GGGTCTTCGGGCTTCAGGTTA-3′	
ALP	NM_013059.1	Forward 5′-TGGTACTCGGACAATGAGATGC-3′	219
		Reverse 5′-GCTCTTCCAAATGCTGATGAGGT-3′	
*β*-Actin	NM_031144	Forward 5′-TGCTATGTTGCCCTAGACTTCG-3′	240
		Reverse 5′-GTTGGCATAGAGGTCTTTACGG-3′	

## Abbreviations

AR: * Actaea racemosa*
BMSCs: Bone marrow mesenchymal stem cells
ALP: Alkaline phosphatase
cGMP: Cyclic guanosine monophosphate
NOS: Nitric oxide synthase
NO: Nitric oxide
PMOP: Postmenopausal osteoporosis
OPG: Osteoprotegerin
OC: Osteocalcin
ER: Estrogen receptor
HUST: Huazhong University of Science and Technology
L-NAME: L-Nitro arginine methyl ester
CTL: Control.
